# Gut microbiome diversity and function during hibernation and spring emergence in an aquatic frog

**DOI:** 10.1371/journal.pone.0298245

**Published:** 2024-02-16

**Authors:** Ji-Eun Lee, Jun-Kyu Park, Yuno Do

**Affiliations:** Department of Biological Sciences, Kongju National University, Gongju, Republic of Korea; Stockholm University, SWEDEN

## Abstract

The gut microbiota maintains a deeply symbiotic relationship with host physiology, intricately engaging with both internal (endogenous) and external (exogenous) factors. Anurans, especially those in temperate regions, face the dual challenges of significant external influences like hibernation and complex internal variances tied to different life histories. In our research, we sought to determine whether different life stages (juvenile versus adult) of the Japanese wrinkled frog (*Glandirana rugosa*) lead to distinct shifts in gut bacterial communities during winter (hibernation) and its subsequent transition to spring. As hypothesized, we observed a more pronounced variability in the gut bacterial diversity and abundance in juvenile frogs compared to their adult counterparts. This suggests that the gut environment may be more resilient or stable in adult frogs during their hibernation period. However, this pronounced difference was confined to the winter season; by spring, the diversity and abundance of gut bacteria in both juvenile and adult frogs aligned closely. Specifically, the variance in gut bacterial diversity and composition between winter and spring appears to mirror the frogs’ ecological adaptations. During the hibernation period, a dominance of Proteobacteria suggests an emphasis on supporting intracellular transport and maintaining homeostasis, as opposed to active metabolism in the frogs. Conversely, come spring, an uptick in bacterial diversity coupled with a dominance of Firmicutes and Bacteroidetes points to an upsurge in metabolic activity post-hibernation, favoring enhanced nutrient assimilation and energy metabolism. Our findings highlight that the relationship between the gut microbiome and its host is dynamic and bidirectional. However, the extent to which changes in gut bacterial diversity and composition contribute to enhancing hibernation physiology in frogs remains an open question, warranting further investigation.

## Introduction

The interplay between an animal’s gut microbiota and its physiology has become an area of intense scrutiny over recent years, shedding light on a relationship refined through intricate symbiosis and millions of years of co-evolution [1]. These complex symbiotic associations, known as holobionts, are a testament to the evolutionary and adaptive strategies that have shaped countless species [2]. The relationship between an organism and its microbiota is more than a mere ecological coincidence; it is a finely tuned partnership forged through evolutionary pressures [3–5]. Within this vibrant microbial landscape, the gut microbiome—comprising bacteria, fungi, and viruses—engages in a dynamic interaction with enterocytes, the primary cells lining the gut [6]. This relationship is crucial for maintaining the integrity and homeostasis of the gut environment. Beyond digestion, the gut microbiota contributes to a range of physiological processes, acting as auxiliary metabolic organs [7].

Factors shaping the gut microbiota can be broadly classified into endogenous and exogenous influences. Endogenous factors include the host’s genetics, physiology, behavior, and age [6,8]. Exogenous factors encompass diet, medications, environmental pollutants, and other external stimuli [9,10]. Although a considerable amount of research has been conducted on endothermic (’warm-blooded’) animals, there exists a significant knowledge gap concerning ectothermic (’cold-blooded’) species, such as amphibians, fish, and insects [11]. Among ectotherms, amphibians—and frogs, in particular—present a compelling case for the study of gut microbial dynamics [12,13]. Frogs are especially sensitive to environmental changes and possess a complex life history, making them exceptional models for research on gut microbiota [14]. The gut microbiome of frogs plays a critical role in maintaining homeostasis, and its disruption can have profound implications. We highlighted how changes in the frog’s gut microbiome are not only associated with individual disease infections but also interact with various external factors, potentially leading to increased susceptibility to lethal pathogens. This aspect of research into the gut microbiota dynamics of frogs is especially significant, as it offers valuable insights into predicting the health of the host and understanding the mechanisms underlying disease development [15,16]. Our expanded discussion underscores the importance of studying gut microbiomes in amphibians for broader applications in health and disease research.

A notable aspect of frogs, central to our study, is their ectothermic nature, which significantly influences their physiology, behavior, and ecology, particularly in relation to temperature variations in temperate zones. This becomes highly relevant in temperate zones where seasonal changes lead to temperature-driven adaptations such as hibernation. During hibernation, frogs experience a sharp decline in metabolic rates and cease feeding activities, thereby entering a state of prolonged fasting [17]. These physiological adjustments could have far-reaching consequences on their gut microbiota [13]. Moreover, the life stages of frog post-metamorphosis—juveniles and adults—differ in their energy investments; juveniles prioritize growth, while adults focus on reproduction [18,19]. In light of previous research findings, including Tong Q et al.’s study, we note that the gut bacterial composition between juvenile and adult frogs generally does not show significant variation within a single season [12,20]. Our study extends this understanding by examining how these microbial communities respond to seasonal changes, potentially influenced by differences in life stages. This consideration adds depth to our exploration of gut microbiota complexity in frogs [12].

In our study, we focus on the Japanese wrinkled frog (*Glandirana rugosa*), a species known for its unique aquatic hibernation behavior. These aquatic hibernating frogs predominantly reside in flowing streams with high dissolved oxygen levels and shallow depths. They endure the winter by navigating along oxygen concentration gradients and employing a skin-based gas exchange strategy [17]. Throughout their extended aquatic hibernation in winter, they may undergo alterations in osmotic pressure and ionic balance due to overhydration [14]. The frogs’ capacity to hibernate underwater requires a suite of adaptations to manage risks such as freezing, dehydration, and hypoxia.

The composition of the frog gut microbiota is known to undergo dramatic changes during metamorphosis, as documented in the literature [8,21]. Furthermore, the developmental stage of the host plays a crucial role in shaping the diversity and functional profile of the gut bacterial community. Additionally, the various life stages following metamorphosis may exhibit significant responses to the gut microbiota, necessitating further investigation.

We hypothesize that the life stages of frogs, particularly the difference between juveniles and adults, combined with their unique physiological adaptations during hibernation, lead to distinct shifts in the composition of their gut microbiota. Such insights could provide a better understanding of the microbiome’s resilience and adaptability across different life stages and environmental conditions [22].

## Method & material

### Animal collection

The Japanese wrinkled frog (*G*. *rugosa*), endemic to East Asia, is notable for its underwater hibernation [23]. This species remains consistent in its habitat from the onset of hibernation through breeding, facilitating its collection during both hibernation and post-hibernation periods. Frogs were hand-captured from water under a shaded bridge at three locations in Gongju-si, Chungcheongnam-do, Korea, ensuring minimal stress and disturbance to the animals. In total, 20 adults (10 in winter, 10 in spring) and 20 juveniles (10 in winter, 10 in spring) were captured between January and February 2022 (winter/hibernation) and April and May 2022 (spring/post-hibernation). Once captured, frogs were transported to the Animal Laboratory at Kongju National University for gut bacterial analysis. Each frog’s snout-vent length (SVL) and weight were measured using a digital caliper.

For euthanization, we employed a bath solution of tricaine methane sulfonate (MS-222) combined with sodium bicarbonate at a pH of 7.0. Given the recommended concentration range of 0.2 g/L to 5.0 g/L for amphibian anesthesia [24,25], we utilized 5 g/L for adults and 3 g/L for juveniles to ensure a humane and stress-free procedure [26–28]. A frog was deemed euthanized upon observing the loss of reflexes and respiratory and cardiac arrest [24,25]. The euthanasia process typically took about 5 minutes per frog, using MS-222. Subsequently, to maintain the integrity of the samples, the dissection for harvesting the frog’s large intestines was completed within a maximum of 30 minutes post-euthanasia. The dissection was performed under stringent sterile conditions, which involved flame sterilization, alcohol sterilization, and sterile water washing, to minimize the risk of sample cross-contamination and ensure the reliability of our findings. Gut samples were individually stored in sterilized 2 ml microtubes at −80°C until subsequent analysis. In our contamination control measures, we included negative controls consisting of distilled water, gloves, scissors, tweezers, and other equipment, each swabbed with a cotton swab. These controls were subjected to the same DNA extraction and PCR protocols as our biological samples. Our rigorous analysis confirmed the absence of DNA extraction and PCR amplification in these negative controls, indicating negligible contamination risk. Consequently, we determined that a separate analysis of the negative control group was not required, underlining the reliability of our experimental results. All animal procedures were executed in strict accordance with ethical guidelines and received approval from the institutional review board at Kongju National University (KNU_2022–01).

### Microbial DNA extraction and 16S amplicon sequencing

Lysis buffer was added to the samples, after which they were homogenized at a speed of 30m/s using a TissueLyser II (Qiagen, Hilden, Germany) for a duration of five minutes. The genomic DNA of large intestinal bacteria from frogs was extracted using a Qiagen DNeasy Powersoil Pro Kit (Qiagen, Hilden, Germany). The resulting DNA was quantified with a Qubit fluorometer (Denovix Inc., Wilmington, DE, USA) using the QFX dsDNA High Sensitivity Assay Kit (Denovix Inc., Wilmington, DE, USA). The DNA quality was verified via 1% electrophoresis, confirming that no samples were abnormal. This genomic DNA served as the template in PCR amplifications that targeted the V4 region of the 16S rRNA genes, using the Illumina overhang adapter sequences F515 (5’-CACGGTCGKCGGCGCCATT-3’) and R806 (5’-GGACTACHVGGGTTWTCTAAT-3’). The resultant PCR product was purified using Agencourt AMPure XP (Beckman Coulter, Brea, CA, USA). Index PCR was subsequently conducted with the Nextera XT Index Kit (Illumina, San Diego, CA, USA). After a second round of purification, all samples were standardized to a concentration of 10 nmol/L. A pooled library was prepared and further diluted to 1 nmol/L. This was sequenced on an Illumina MiniSeq system (Illumina, Inc., San Diego, CA, USA).

### Bioinformatic analysis

To analyze the gut bacterial community of the frogs, the resulting sequencing data was converted into a FASTQ format, after which paired-end sequencing reads were processed using QIIME2 (software version 2023.5) [29]. Chimera sequences were eliminated via the DADA2 plug-in [30]. The disordered nucleotide sequences were arranged for streamlined analysis. Bacterial species were clustered into OTUs (operational taxonomic units) with a 97% similarity threshold. Each OTU’s representative sequences were annotated, with clustering information derived from an algorithm referencing the 16S EzBioCloud database (https://www.ezbiocloud.net/, accessed on June 10, 2023). Analyses of alpha and beta diversity, as well as taxonomic assessments, were facilitated using the microeco package (version 0.20.0) in R software (version 4.3.1) [31]. Functional gene predictions, based on species composition, were analyzed using the Tax4Fun functional classification database (http://tax4fun.gobics.de, accessed on June 10, 2023), which classified OTUs according to sequence similarity [32].

### Statistical analysis

The microeco package (version 0.20.0) [31] in R software (version 4.3.1) was employed to compute and analyze both alpha and beta diversity metrics. Alpha diversity comparisons in the gut bacterial community were based on observed species, the Chao1 index, the Shannon index, and the phylogenetic distance (PD) whole tree index. For bacterial taxonomic data, a Hellinger transformation was performed. This data was then contrasted with a dissimilarity matrix constructed using the Bray-Curtis distance metric. A permutational multivariate analysis of variance (PERMANOVA) was actually conducted to assess differences in gut bacterial composition, correctly reflecting the analysis performed. The results were visualized using PCoA, based on the Bray-Curtis dissimilarity matrix. To discern significant differences in the alpha and beta diversity of the gut bacterial community, the Kruskal-Wallis test, followed by Dunn’s post hoc test, was executed using JASP (version 0.17.3) [33]. Linear Discriminant Analysis (LDA) was conducted as part of the LEfSe (LDA Effect Size) analysis to identify significantly different gene functional taxa across groups. The LDA scores reflect the degree of difference in the relative abundance of these taxa. In this process, adult frogs during spring were used as the reference group. We employed a non-parametric Kruskal-Wallis test for initial detection of features with different abundances, followed by Wilcoxon rank-sum tests for pairwise group comparisons. The final LDA scores indicate the taxa’s contribution to the differentiation between groups, with higher scores signifying a more substantial impact. Our results, displayed in a ranked format, highlight the key functional taxa that differentiate each group from the spring adult frogs. All findings were deemed statistically significant at *p* < 0.05.

## Result

### Physical comparison of Japanese wrinkled frogs

There were notable differences in body length (H = 29.466, *p* < 0.001) and weight (H = 29.399, *p* < 0.001) across the four groups (Table 1). These differences in body length and weight were more attributed to life stages (Dunn’s post hoc, *p* < 0.05) than to seasonal changes (Dunn’s post hoc, *p* > 0.05). Upon reassessment, we have found that there was no significant change in the overall body condition, a more integrative measure of physical health, for both juvenile and adult frogs from winter to spring.

**Table 1:** Comparison (mean ± standard deviation) of length and weight during (winter) and after (spring) hibernation in adult and juvenile Japanese wrinkled frogs, aquatic overwintering frogs. The significant differences (*p* < 0.05) were determined using Dunn’s post hoc test after a Kruskal-Wallis test, and are represented by lowercase letters.

Parameter	Adult		Juvenile	
	Winter	Spring	Winter	Spring
**SVL (mm)**	70.58±9.61 ^a^	69.09±11.21 ^a^	30.34±1.10 ^b^	30.06±1.61 ^b^
**Weight (g)**	5.02±2.61 ^a^	5.41±2.47 ^a^	1.88±0.32 ^b^	1.62±0.40 ^b^

### Alpha diversity of the gut bacterial communities in Japanese wrinkled frogs

In the comprehensive analysis of the gut microbiota through 16S amplicon sequencing, we observed significant variations in microbial diversity across different groups. The sequencing effort yielded a total of 243,744 reads. In the winter adult frog group, we identified a substantial microbial diversity with 303,439 reads, resulting in 40,391 Operational Taxonomic Units (OTUs). This diversity suggests a complex microbial ecosystem during the winter season for adult frogs. Comparatively, the winter juvenile frog group presented a slightly different profile with 237,214 reads and 37,051 OTUs. This indicates a distinct microbial composition in juvenile frogs, potentially reflecting developmental differences in the gut microbiome at this life stage. Notably, the spring juvenile frog group showed a marked increase in microbial diversity, with the sequencing results indicating 251,041 reads and an impressive 94,292 OTUs. This significant rise in OTUs underscores the dynamic nature of the gut microbiota, responding to both the developmental stage and seasonal changes. Similarly, in the spring adult frog group, we observed a high level of microbial diversity, indicated by 323,263 reads and 69,070 OTUs.

**Fig 1:**
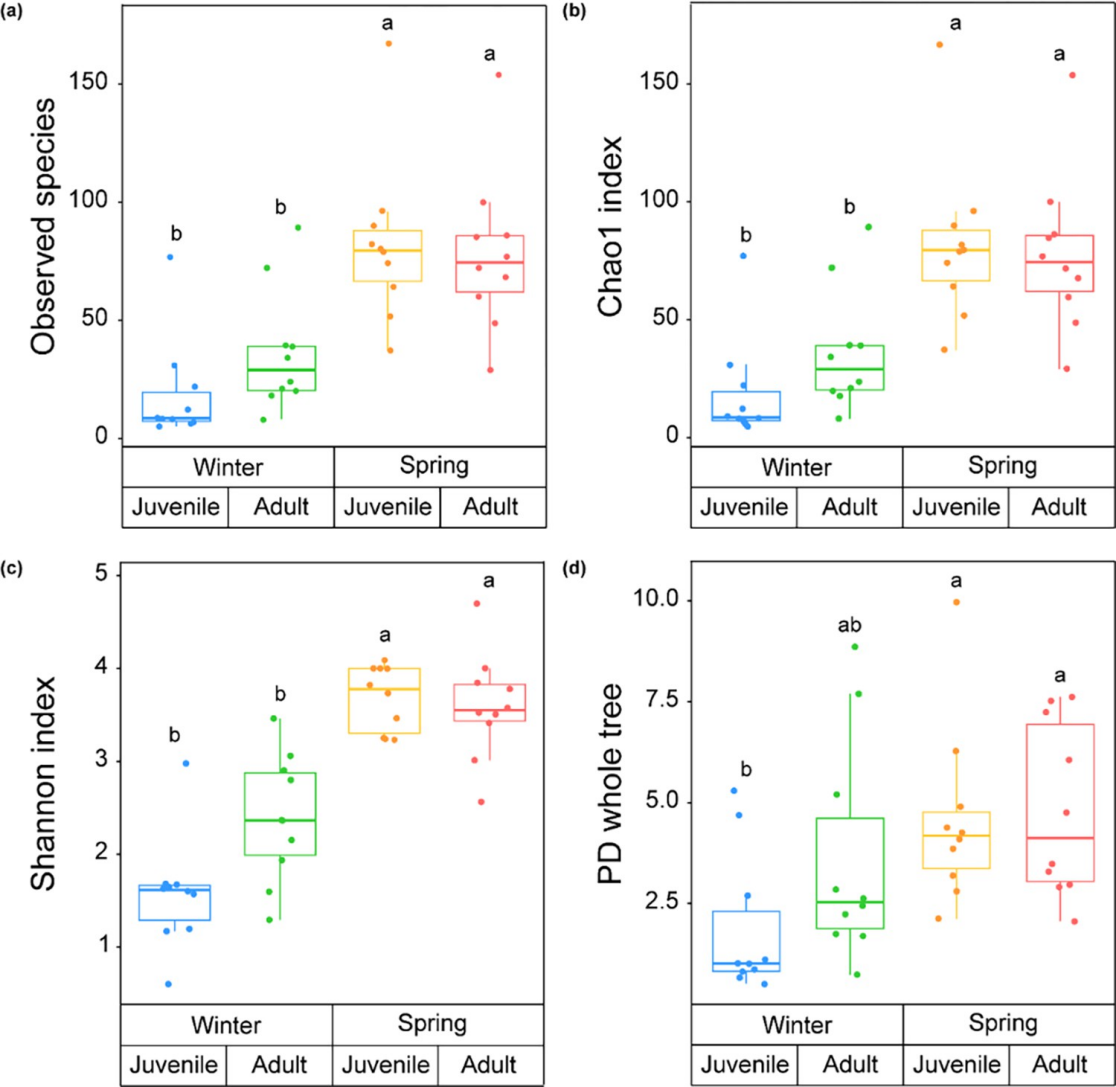
Comparison in alpha diversity of gut bacterial community from adult and juvenile frogs during (winter) and after (spring) hibernation: (a) Observed species, (b) Chao1 index, (c) Shannon index, and (d) phylogenetic diversity (PD) whole tree. The thick line in the middle represents the median, the top and bottom of the box represent the third and first quartiles, respectively, the ends of the lines represent the 1.5 interquartile range, and dots represent individual samples. Significance of differences (*p* < 0.05) was attributed to Dunn’s post hoc test after Kruskal-Wallis test and indicated by lowercase letters.

Distinct differences were observed in the Observed species (H = 21.369, *p* < 0.001) and Chao1 index (H = 21.369, *p* < 0.001), indicators of bacterial community OTU abundance, across the four groups, encompassing both seasonal and life stage variations. The Shannon index (H = 28.064, *p* < 0.001), signifying bacterial community evenness, and the PD whole tree (H = 11.445, *p* < 0.001) also displayed significant differences across these groups. There was no discernible difference in Observed species, Chao1 index, or Shannon index based on life history (Dunn’s post hoc, *p* > 0.05). However, seasonal changes were significant contributors to variations in alpha diversity among groups (Fig 1A–1C). Contrastingly, the PD whole tree index revealed variations due to seasonal changes only in juvenile frogs (Fig 1D). In essence, juvenile frogs displayed a more pronounced variation in alpha diversity than adults from winter to spring.

### Beta diversity of the gut bacterial communities in Japanese wrinkled frogs

**Fig 2:**
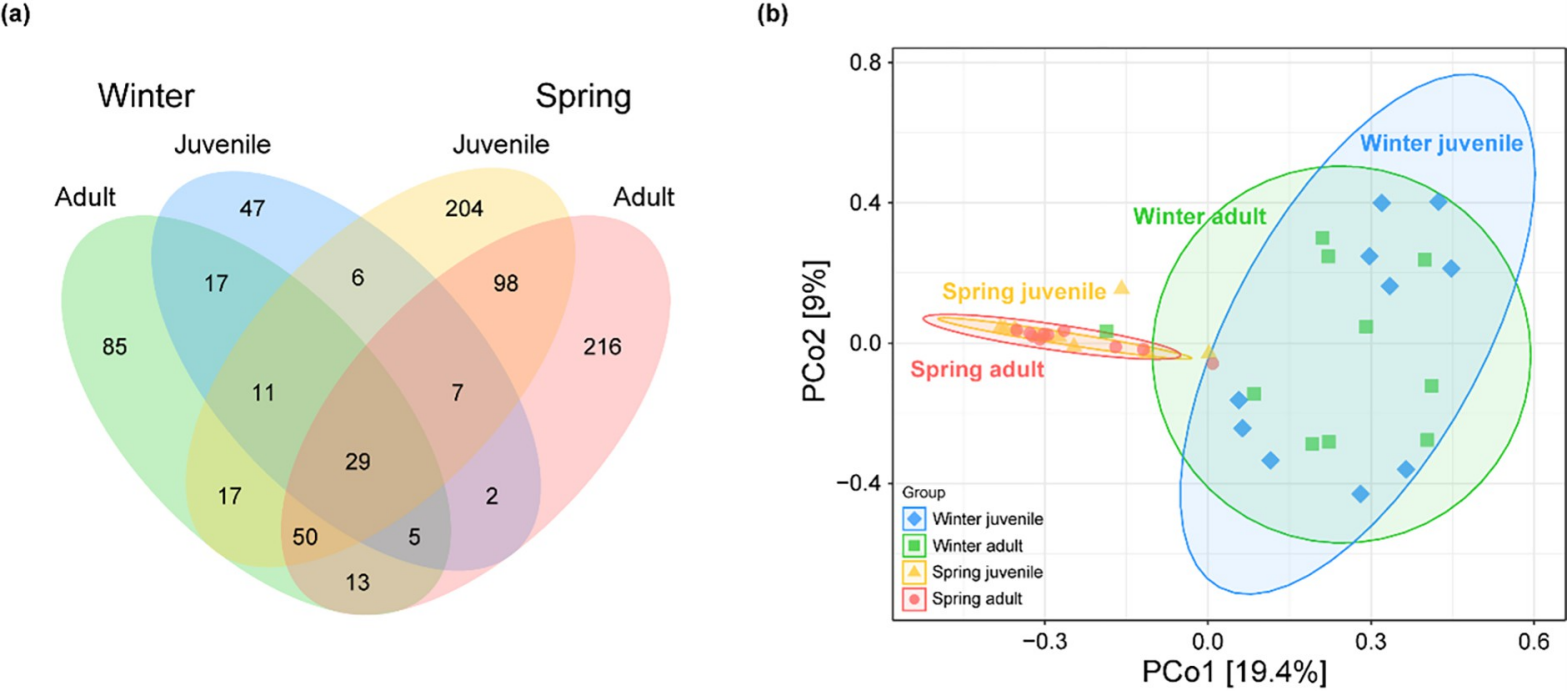
Differences in gut bacterial structure from adult and juvenile frogs during (winter) and after (spring) hibernation: (a) Venn diagram showing gut bacterial OTUs of adults and juveniles of frog from during (winter) and after (spring) hibernation. The number indicated where the circles overlap is the number of shared OTUs by the groups, and OTUs to one group are called unique OTUs. (b) PCoA based on the Bray-Curtis dissimilarity of the gut bacterial composition in adults and juvenile frogs from during (winter) and after (spring) hibernation.

The Venn diagram showed 29 shared OTUs across all four groups (Fig 2A). Unique OTUs in adult frogs during the winter were approximately double those in juveniles. The unique OTUs from winter to spring increased by 157 for juvenile frogs and 131 for adult frogs. However, in the spring, there was no notable difference in OTU numbers between the two groups. Especially in juveniles, there was a more pronounced shift in unique OTUs with the changing seasons than in adults. For shared OTUs, 62 were found between both juvenile and adult frogs during the winter, while the spring showed 184 shared OTUs. Between winter and spring, 97 OTUs were shared among adults and 53 among juveniles (Fig 2A). These findings suggest that seasonal shifts had a more significant impact on the gut bacterial community composition in juvenile frogs compared to adults.

A PERMANOVA revealed a significant difference in gut bacterial composition among the four groups (F = 3.1942, *p* < 0.001). A subsequent PCoA illustrated clear separations between the gut bacterial communities of frogs during winter and spring. PCo1, accounting for 19.4% of the variance, effectively differentiated the communities between seasons. On the other hand, PCo2, representing 9% of the variance, didn’t show such segregation (Fig 2B). Thus, the primary factor in gut bacterial community differentiation was the seasons rather than life stages.

### Analysis of relative abundance and composition of the gut bacteria

**Fig 3:**
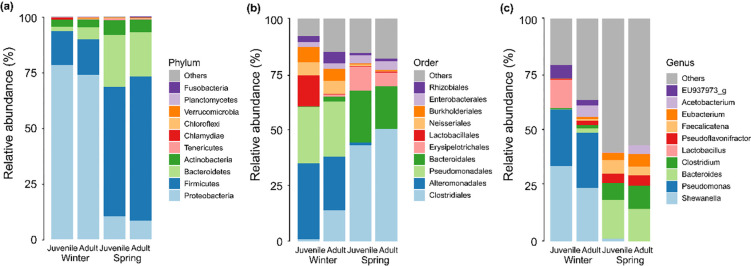
Relative abundance of gut bacterial community from adult and juvenile frogs during (winter) and after (spring) hibernation: (a) at the phylum level in the four groups (b) at the order level in the four groups (c) at the genus level in the four groups.

In the gut of adult and juvenile frogs collected during winter, Proteobacteria was the predominant phylum (75.97%), followed by Firmicutes (15.57%) and Bacteroidetes (3.68%) (Fig 3A). Conversely, in frogs collected during spring, Firmicutes dominated at 61.39%, with Bacteroidetes (21.58%) and Proteobacteria (9.39%) following. Significant differences were observed in the abundance of Proteobacteria (H = 24.858, *p* < 0.01), Firmicutes (H = 21.522, *p* < 0.01), and Bacteroidetes (H = 18.444, *p* < 0.01) among the four groups, unlike other bacterial communities. While the gut bacterial abundance was similar between adult and juvenile frogs in both seasons (Dunn’s post hoc, *p* > 0.05), Proteobacteria was notably more abundant in winter. Conversely, spring saw a rise in Firmicutes and Bacteroidetes (Dunn’s post hoc, *p* < 0.05).

At the order level, Clostridiales was most abundant (46.84%) in frogs during spring, followed by Bacteroidales (21.11%) and Erysipelotrichales (8.48%) (Fig 3B). In winter, Pseudomonadales led at 25.02%, trailed by Alteromonadales (21.98%) and Neisseriales (5.91%). Significant differences among the four groups were observed in the abundance of Clostridiales (H = 26.981, *p* < 0.001), Alteromonadales (H = 16.429, *p* < 0.001), Pseudomonadales (H = 30.689, *p* < 0.001), and Bacteroidales (H = 24.494, *p* < 0.001). Pseudomonadales and Alteromonadales peaked in winter, whereas spring favored Clostridiales and Bacteroidales (Dunn’s post hoc, *p* < 0.05). Lactobacillales (H = 8.549, *p* = 0.036) stood out, showing a relatively high abundance of 13.73% exclusively in juvenile frogs during spring (*p* < 0.05).

At the genus level, winter saw dominance by *Shewanella* (28.98%) and *Pseudomonas* (24.99%) in the gut of both adult and juvenile frogs (Fig 3C). *Bacteroides* marked the third-highest abundance in adult winter frogs (2.03%), whereas juvenile winter frogs displayed a third-place prominence for *Lactobacillus* (12.81%). Conversely, spring frogs showcased *Bacteriodes* as dominant (16.03%), followed by *Clostridium* (8.98%) and *Faecalicatena* (5.00%). In this season, adult frogs had *Eubacterium* (5.57%) as the third most abundant, while juvenile frogs favored *Faecalicatena* (6.10%). Abundances of *Shewanella* (H = 16.429, *p* < 0.001), *Pseudomonas* (H = 31.778, *p* < 0.001), and *Bacteroides* (H = 23.817, *p* < 0.001) remained consistent across seasons (Dunn’s post hoc, *p* > 0.05). Notably, overwintering frogs displayed heightened levels of *Shewanella* and *Pseudomonas*, while *Bacteroides* thrived post-hibernation (Dunn’s post hoc, *p* < 0.05). However, only the overwintering juvenile frogs showed a pronounced presence of *Lactobacillales* (H = 13.045, *p* = 0.005) and *Clostridium* (H = 11.303, *p* = 0.010) (*p* < 0.05).

### Analysis of function prediction of the gut bacteria

Differences in the relative abundance of functional taxa between winter and spring were evident, but these differences were not observed between adult and juvenile frogs. At Level 1, Metabolism was the most predominant function observed in the gut bacterial communities for both the winter and spring groups. Following this, in descending order, were Environmental Information Processing, Genetic Information, Cellular Processes, Human Diseases, and Organismal Systems found across all four groups. Notably, the functional taxa of Metabolism and Environmental Information Processing were more prominent in the spring group compared to the winter group.

**Fig 4:**
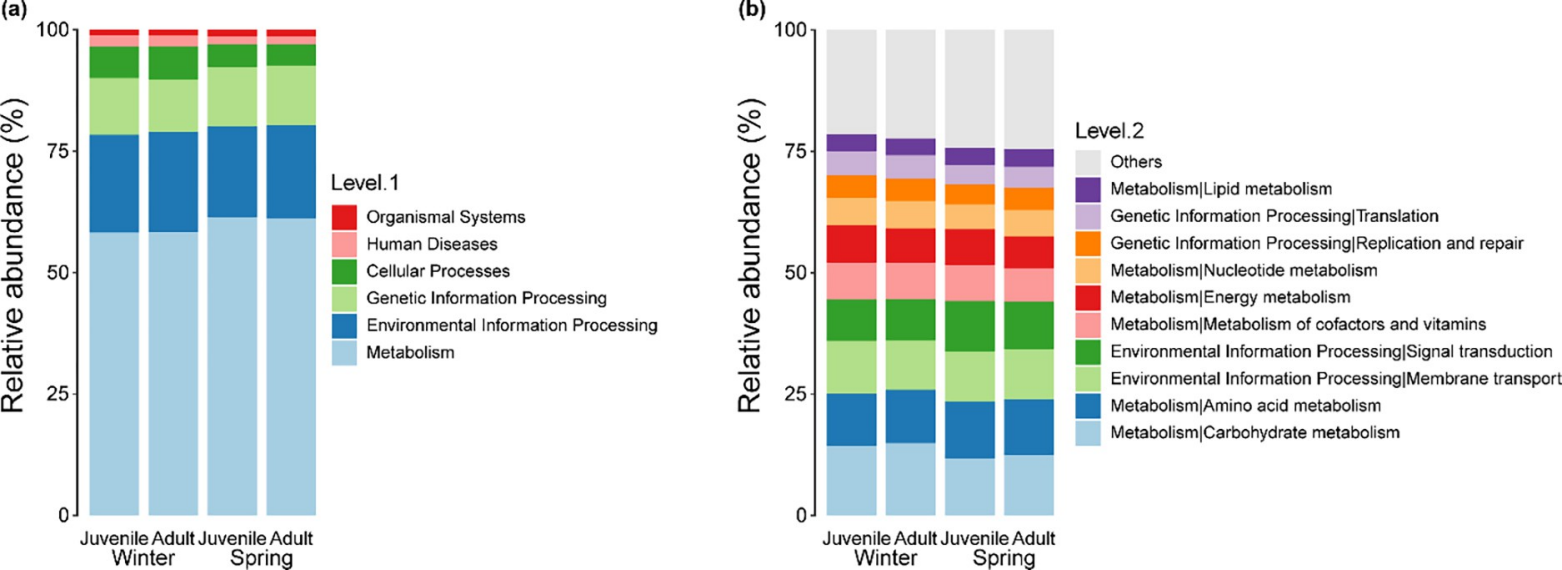
Relative abundance of annotated functional groups in gut bacterial community from adult and juvenile frogs during (winter) and after (spring) hibernation: (a) Top level of relative abundance in functional groups (b) second level of relative abundance in functional groups.

At Level 2, the Carbohydrate Metabolism function was most pronounced in both the winter and spring groups. Subsequently, in descending order, came Amino Acid Metabolism, Environmental Information Processing Membrane Transport, Environmental Information Processing Signal Transduction, Metabolism of Cofactors and Vitamins, and Energy Metabolism (Fig 4B). The spring group, in particular, exhibited a stronger focus on Carbohydrate Metabolism, Amino Acid Metabolism, and Signal Transduction functions compared to the winter group.

**Fig 5:**
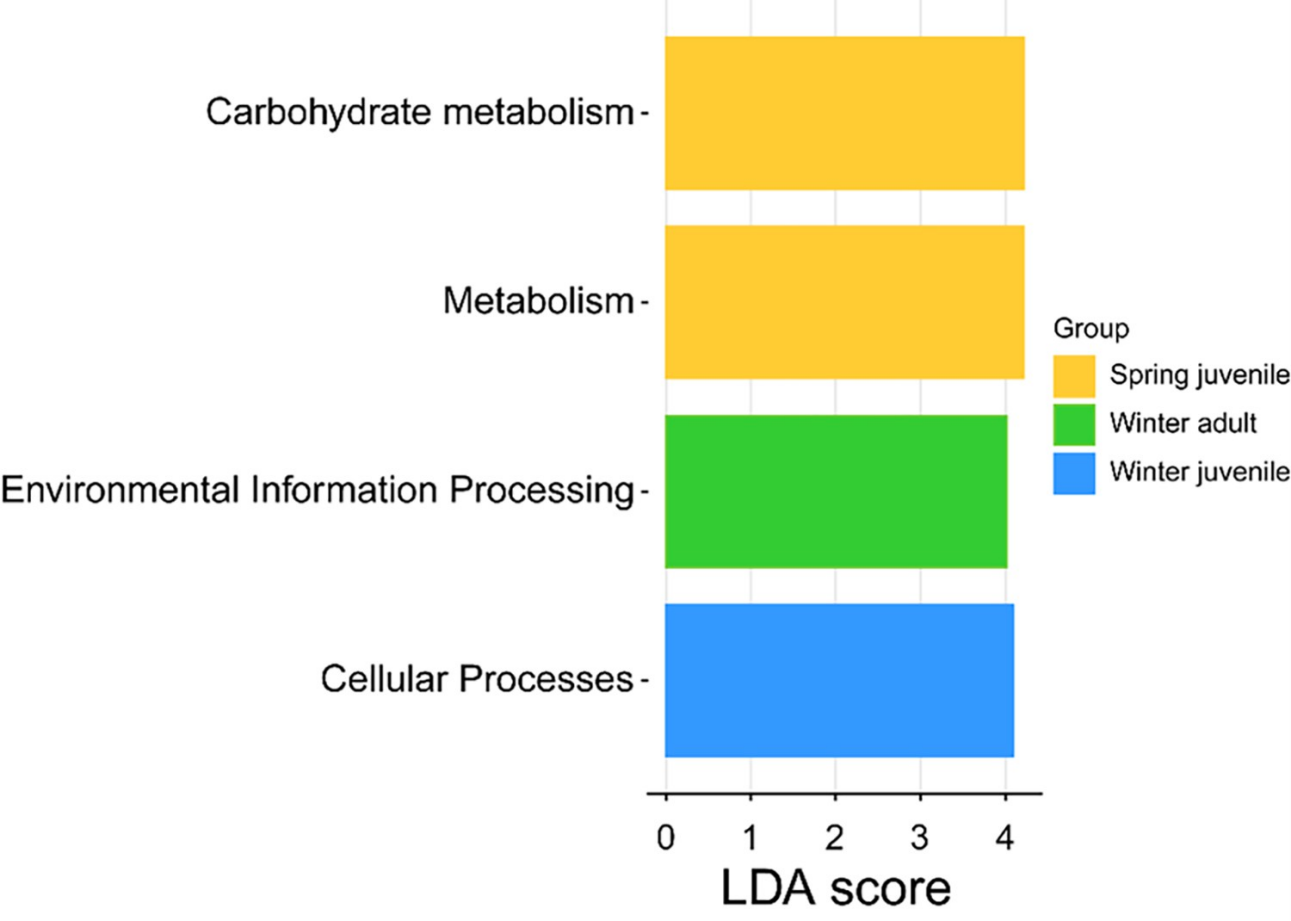
Linear discriminant analysis (LDA) score reflecting the difference in relative abundance after Linear discriminant analysis Effect size (LEfSe) based on adult frog in spring. The histogram of LDA score showed the gene functional taxa, whose threshold was set 4.0.

A linear discriminant analysis (LDA) was conducted to contrast the relative abundances of gut bacteria at different taxonomic levels across the four groups (Fig 5). The results revealed that Cellular Processes dominated in juvenile frogs during hibernation. In contrast, adult frogs during hibernation exhibited a high enrichment of the Environmental Information Processing function. Post-hibernation, juvenile frogs displayed an abundance of Metabolism and Carbohydrate Metabolism functions. The LDA further showed a decline in Cellular Processes and Environmental Information Processing functions in the spring—functions that were enriched during the winter. Simultaneously, there was a rise in Metabolism and Carbohydrate Metabolism functions.

## Discussion

### Gut bacterial diversity of juvenile and adult frogs during and after hibernation

This fluctuating diversity in the gut microbiome could potentially enhance the metabolic activities or response strategies of frogs during their hibernation phases, reflecting the bidirectional interaction between the host and its microbiome. Overwintering aquatic frogs primarily adapt to cold temperatures during hibernation, but they also encounter other environmental challenges, such as fluctuations in oxygen gradients [17]. Upon emerging from hibernation in the warmer spring months, they encounter a broader spectrum of environmental challenges than in winter. Depending on their post-hibernation physical state, some frogs might wake up earlier, begin feeding sooner, or even migrate to different habitats [34–36]. In such scenarios, a diverse gut microbiome could contribute to population resilience by potentially enhancing metabolic adaptability post-hibernation [37]. This increased microbial diversity, possibly a response to environmental shifts, may support post-hibernation growth and reproduction in both juvenile and adult frogs. We postulate a mutual influence between the frog’s adaptation and the transitions in its gut microbiome.

Earlier studies indicated minimal differences in the gut microbiomes of adult and juvenile frogs during summer [12]. In contrast, our research shows that juveniles display more distinct changes in gut bacterial diversity than adults during hibernation. Such variations hint that the influences of life history on the gut bacterial community might be more prominent during hibernation, differing across seasons. In spring, both adult and juvenile frogs showcased similar levels of gut bacterial diversity. Yet, in winter, juveniles had a reduced bacterial diversity, as evidenced particularly by the PD diversity index. The prominent variability in juveniles might stem from distinct microbial compositions between spring and winter. From an ecological perspective, stability is assessed by low variability (or high resistance) and by the resilience provided by strong biodiversity [38,39]. Consequently, a decline in diversity or an increase in variability in the gut microbiome could signify potential instability within the host’s gut ecosystem [37,40]. Given their smaller size and the challenges of undergoing their initial metamorphosis and hibernation, juvenile frogs may face greater instability during hibernation. However, by spring, it appears that the gut ecosystems of the frogs regain a level of microbial diversity comparable to that observed in adult frogs. This observation suggests that although juvenile frogs may face greater risks in winter, they attain a gut bacterial composition by spring similar to that of adults, which might be significant for their resilience to seasonal changes. While our study provides insights into the gut microbiome during and post-hibernation, it does not include data on the pre-hibernation period. Future studies are needed to track microbiome changes throughout the entire season, including the critical pre-hibernation phase.

### Composition and functional groups of gut bacterial community during and after hibernation in frogs

The gut bacterial community of frogs undergoes seasonal compositional shifts, influenced by hibernation and life history transitions [12]. Seasonal fluctuations are the primary drivers of these variations, more so than life history differences. Proteobacteria emerges as the dominant bacterial group in both juvenile and adult frogs during winter. Found commonly among facultative anaerobes in freshwater environments, this bacterial group flourishes when oxygen levels peak [41]. Aquatic amphibians hibernating over winter necessitate substantial oxygen concentrations for survival. This need propels them to navigate oxygen gradients and occasionally surface to breathe [17]. Amid hibernation, these amphibians sustain a reduced metabolic rate, converting stored glycogen to glucose in oxygen-rich conditions [42]. Additionally, they adjust osmotic pressure and homeostasis in response to prolonged water immersion [17], which in turn affects their gut bacterial composition.

Our findings underscore an elevated presence of functional groups linked to environmental information processing and cellular processes in hibernating frogs. These entities play a pivotal role in intracellular substance transportation and maintaining homeostasis processes integral to ion and gas balance, energy metabolism, and osmotic regulation during hibernation [43,44].

While Tax4Fun is a valuable tool for functional profiling of microbial communities, primarily based on human and mammalian data, its direct application to amphibian microbiomes requires careful interpretation. Recognizing its widespread use in recent amphibian studies, we employed Tax4Fun in our analysis but with added caution. We acknowledge that the extension of mammalian-based microbial function analysis to amphibians may have inherent limitations, underscoring the need for more specialized research in this area [9,45]. A notable observation was the pronounced environmental information processing functions in adult frogs during winter. In contrast, juvenile frogs showed heightened cellular process functions. Given the extended submersion of aquatic amphibians, they’ve developed mechanisms to mitigate excessive ion loss and overhydration [46]. Differences between juveniles and adults, particularly in their bacterial composition, can be ascribed to their size. Larger-bodied adult amphibians harbor more ions and water due to their expansive surface area, necessitating stringent regulation of intra-cellular substance transport. For juveniles, with their relatively smaller size, maintaining cellular homeostasis becomes paramount. Moreover, during winter, juvenile frogs prominently hosted Lactobacillus, a bacterium known for modulating fasting blood glucose and its antioxidant attributes [47]. Given that juveniles may have lower glycogen reserves than adults, it is conceivable that Lactobacillus might influence their hibernation metabolism, but this relationship warrants further exploration to establish a more definitive connection. However, these insights are initial, warranting a more comprehensive analysis.

Post-hibernation sees a surge in the presence of Firmicutes and Bacteroidetes, bacteria integral to energy storage and metabolism. Firmicutes are associated with enhanced nutrient uptake and fat storage [48], while Bacteroidetes are implicated in food digestion and immune development [49]. The physiological shifts following winter necessitate recovery, with frogs undergoing rejuvenation post-hibernation [17,50]. As adult frogs prepare for reproduction and juveniles focus on growth, it is plausible that these bacterial groups may play a role in supporting their metabolic energy needs, although this remains to be further investigated. By spring, a shared trend among both adult and juvenile frogs is an augmented presence of metabolic bacterial communities, underscoring their pivotal role in meeting post-hibernation energy demands. Particularly for juvenile frogs, these bacterial groups are essential as they redirect energy from consumption to growth [42]. Additionally, the Proteobacteria, dominant during hibernation, are adapted to withstand stringent conditions and utilize oxygen, creating a conducive environment for anaerobes [41]. The decline of Proteobacteria post-hibernation coincides with the emergence of Firmicutes and Bacteroidetes, hinting at the potential preparatory role of Proteobacteria for the succeeding bacterial groups. The dynamic nature of the gut microbiota across various life stages calls for deeper research into the interactions between Proteobacteria, Firmicutes, and Bacteroidetes throughout the frog’s lifespan.

## Conclusion

**Fig 6:**
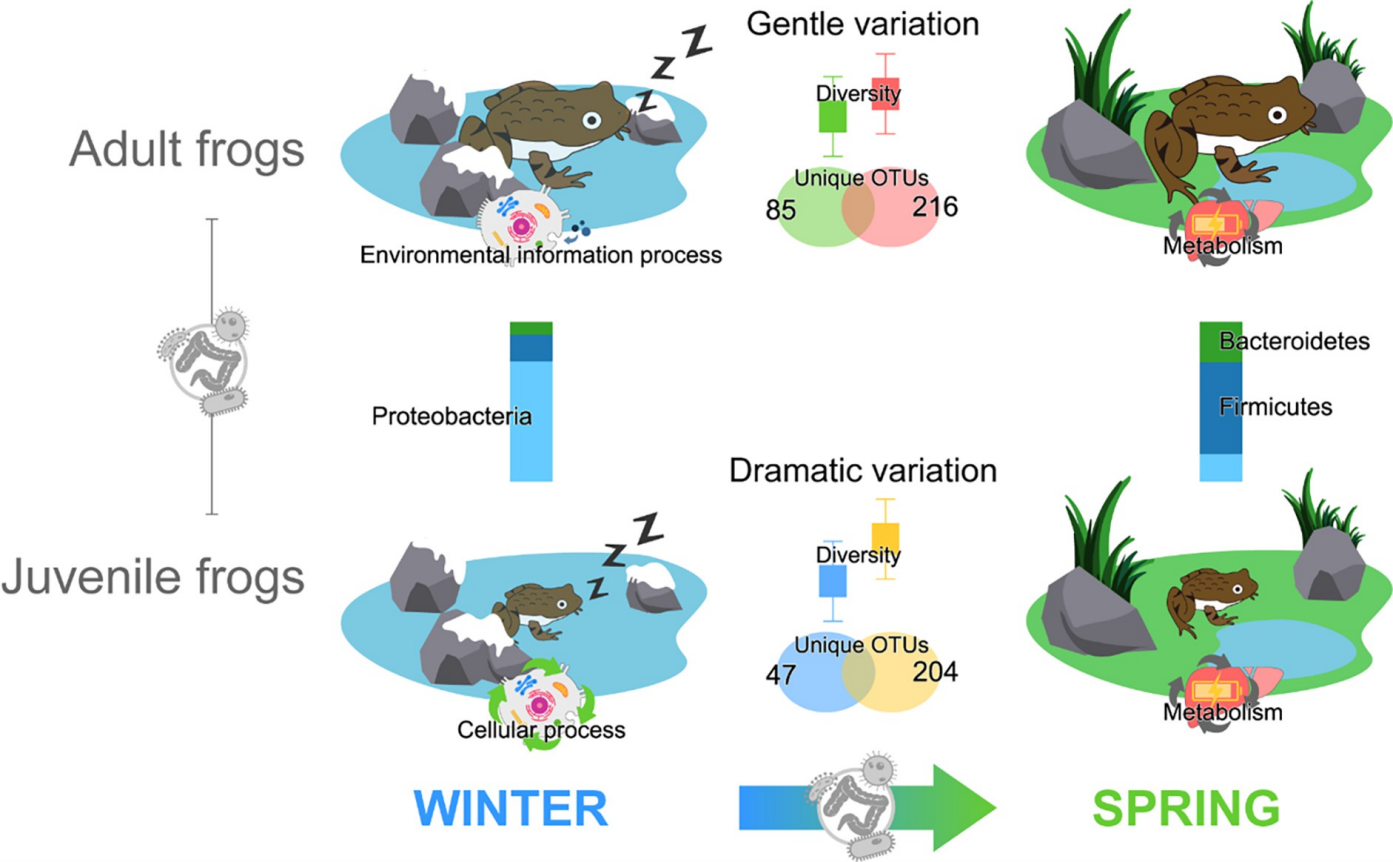
The changes in diversity, composition, and function of the frog gut bacterial community are evident significantly between seasons, and these seasonal differences are greater in juvenile frogs.

The gut bacterial community’s diversity, composition, and function varied significantly during and post-hibernation, with nuances based on the life stage (Fig 6). Adult frogs displayed more stable gut bacterial variations during hibernation. Physical, ecological, and biological factors associated with life history seemingly influence the gut bacterial community’s diversity and stability. It’s vital to recognize that gut microbiota shifts during hibernation can actively support host physiology. To deepen our understanding of host-microbe interactions and hibernation adaptation, we recommend further exploration into how gut microbiota functions align with host physiology. Studies should also focus on bacterial communities affecting current and subsequent life stages, especially connections between Proteobacteria, Firmicutes, and Bacteroidetes. Such re-search will enrich our comprehension of how gut microbiomes bolster physiological processes in ectotherms.
